# Lower-dose prescriptions in the post-marketing situation and the influencing factors thereon

**DOI:** 10.1371/journal.pone.0218534

**Published:** 2019-06-14

**Authors:** Akiko Ogata, Masayuki Kaneko, Mamoru Narukawa

**Affiliations:** Department of Clinical Medicine (Pharmaceutical Medicine), Graduate School of Pharmaceutical Sciences, Kitasato University, Tokyo, Japan; University of California Berkeley, UNITED STATES

## Abstract

The dosage of pharmaceuticals is determined through the process of clinical development and approval review based on clinical trial results; however, the information obtained from clinical trials before approval is limited. Some pharmaceutical products are used at doses lower than those approved for post-marketing use. The aim of this study was to reveal the actual state of lower-dose prescriptions for post-marketing clinical use of pharmaceuticals. We investigated the factors related to the deviation based on therapeutic area, detailed statement of the approved dosage, clinical data package, and post-marketing requirement. Among the new molecular entities approved in Japan between January 2005 and December 2014, we identified products that are orally administered and have the same daily dose for different indications, if any. For these products, we collected information on the actual daily dose from the medical information databases of Medical Data Vision Co., LTD. and JammNet Co., LTD. Products whose dose was lower than the approved dose (maintenance dose excluding the initial dose) in ≥ 30% prescriptions in 2015 were defined here as “lower-dose prescription drugs.” We identified 27 lower-dose prescription drugs out of 113 products investigated. The results of the multivariate analysis revealed that factors related to the Anatomical Therapeutic Chemical classification and the detailed statement of the approved dosage significantly influenced the occurrence of lower-dose prescription, whereas the factors related to clinical data package and post-marketing requirements did not. These results suggest the limitation in determining an optimal dosage for the actual clinical use of a drug based on the information obtained from clinical trials conducted before approval, emphasizing the importance of reexamining the optimal dosage that is applicable to a greater number of patients after marketing, if necessary. We believe that the utilization of real-world data could be of help in this regard.

## Introduction

The determination of drug dosage is one of the important aspects for the effective and safe usage of pharmaceuticals. Generally, the dosage and dosage regimen are decided through the process of clinical development and approval review based on the results of clinical trials. In phase I studies, clinical exposure and tolerability are examined using several doses of a drug in healthy adults and in phase II studies, dose–response relationships are evaluated in a small number of patients, and based on the information obtained, the recommended dose or dose range of the drug is proposed. In phase III studies, the efficacy and safety of the recommended dose are confirmed.

In clinical trials for marketing authorization, there are several restrictions such as exclusion of patients with complications and concomitant medications, and the information obtained from such clinical trials is limited. After marketing, the use of drug is expanded to patients who do not meet clinical trial eligibility criteria, as well as the dose is adjusted according to the condition of individual patients. Thereby, the approved dosage might not be optimal for actual conditions of post-marketing use. It has been reported that the approved dose of approximately 20% of the new molecular entities (NMEs) in the United States between 1980 and 1999 was changed in the post-marketing phase and that the change to a lower dose due to safety issues accounted for approximately 80% of the overall changes [1]. Defined daily dose (DDD), an average daily dose for adults in the primary indication defined by the World Health Organization (WHO), was changed in the post-marketing phase for 115 products between 1989 and 2000, and approximately 60% of them indicated a change to a lower dose [2]. It has been reported that although a lower-dose prescription is often recommended for the elderly population and for reducing side effects [3–6], clinical evidence on using such a low dose is not reflected in the product label (package insert), even if it is published in medical journals [7–8].

To the best of our knowledge, the actual situation of post-marketing prescription of specific products has not been fully investigated so far. Some studies have reported that, in the clinical development process, phase III trials are often performed using doses close to the maximum tolerated dose (MTD) to focus on the efficacy, and the lower doses of a drug are not sufficiently examined [1, 6, 9]. However, there is no evidence supporting the fact that this is the cause of lower-dose prescriptions in the post-marketing phase.

The aim of this study was to reveal the actual state of lower-dose prescriptions in post-marketing clinical use of pharmaceuticals and to investigate the factors that might lead to prescription of drugs at a lower dose. We investigated the actual situation of lower-dose prescriptions by comparing the frequency distribution of the daily dose of each pharmaceutical product using the medical information databases and identified the products for which some prescriptions presented deviation toward lower dose from the approved dosage, and these were termed “lower-dose prescription drugs.”

## Materials and methods

### Drugs examined

Information on the daily dose of 342 pharmaceutical products approved as NMEs in Japan between January 1, 2005 and December 31, 2014 was collected. From the medical information databases we used, only prescription data of daily dose were available; whereas background information for individual patients such as height, weight, and complications was not available, and therefore, we could not correlate them with the prescription data. Therefore, we set inclusion and exclusion criteria to identify pharmaceutical products to be investigated using the medical information databases. The inclusion criteria were as follows: 1) drugs administered orally, 2) drugs indicated for adults, and 3) drugs with the same daily dose for different indications (the daily dose does not differ depending on the indication). The exclusion criteria were as follows: 1) combination drugs, pro re nata (as needed) drugs, and drugs not covered by insurance, 2) drugs with a dosage based on body weight or body surface area, and 3) clinical trials for efficacy and safety not conducted before approval.

### Data sources

In the first survey, we used the medical information databases of the Medical Data Vision Co., LTD. (MDV; Tokyo, Japan) and JammNet Co., LTD. (Tokyo, Japan). The database of MDV contains health claim data and administrative data of hospitals in which the payment was made based on the Diagnosis Procedure Combination/Per-Diem Payment System (DPC/PDPS). The database of JammNet contains medical receipt information from health insurance societies in Japan. We investigated the daily dose of the products prescribed for adults (≥ 15 years old at the time of prescription) between January 1 and December 31, 2015. During the survey period, data from approximately 12.65 million individual patients (12.2% < 15 years old, 45.8% ≥ 15 < 65 years old, and 42.0% ≥ 65 years old) from 225 medical institutions (hospitals only) were included in the database of MDV and data from approximately 630,000 patients (20.3% < 15 years old, 64.0% ≥ 15 < 65 years old, and 15.7% ≥ 65 years old) from 72,156 medical institutions (6544 hospitals and 65,612 clinics) were included in the database of JammNet. Although the database of MDV has a large amount of data equivalent to one in seven Japanese citizens, considering the fact that information from clinics is not included and that the age composition differs between the two databases, we also utilized the database of JammNet to gain further insights on prescription trends.

In the second investigation, we extracted information pertaining to products such as Anatomical Therapeutic Chemical (ATC) classification, detailed statement of the approved dosage, clinical data package, and post-marketing requirement from the publications, including product label, approval submission dossier (Common Technical Document: CTD), and review report.

### Definition of lower-dose prescription drugs

We counted the prescriptions in each category of daily dose, and calculated the percentage to the total number of prescriptions in the two databases. The categories were as follows:

1. In Japanese product labels, approved dose described in the section “Dosage and administration” and dose information for special populations described in the section “Precautions concerning dosage and administration” are clearly distinguished [10]; therefore, the dose for special populations was compiled separately from the approved doses, if any.
2. The initial dose and titration dose were defined as “initial dose” and compiled separately from the maintenance dose because the initial dose is prescribed only for a limited period before reaching the maintenance dose.
3. If there was a separate statement on the initial dose for special populations in the product label, it was compiled as a different category.
4. Categories with doses less than the minimum dose and exceeding the maximum dose mentioned in points 1 to 3 above were also set respectively, if any.

Products whose percentage of prescriptions corresponding to the category of doses lower than the approved dose, or the approved maintenance dose if the initial dose is set, was ≥ 30% in the database of either MDV or JammNet were defined as “lower-dose prescription drugs”. We set this threshold value as ≥ 30% considering the proportion of elderly people (26.0%) in Japan as of January 2015 [11], which indicates that the drug was prescribed to a large number of patients. In addition, in order to perceive the distribution of the daily dose for each drug, we calculated the median and quartile points using the database of MDV.

### Factors examined

To explore the factors influencing lower-dose prescription drugs, we extracted information pertaining to ATC classification, detailed statement of the approved dosage, clinical data package, and post-marketing requirement. Ten factors investigated in the present research were as follows:

1. ATC classification. Using the ATC code, products of “L01 antineoplastic agents” were classified as antineoplastic agents, because they have characteristics considerably different from those of other pharmaceutical products in terms of dosage selection in the clinical development process [12].
2. Detailed statement of the approved dosage. Three factors, “dose in range,” “maintenance dose different from the initial dose,” and “upward/downward dose adjustment,” were investigated. “Dose in range” indicates that the approved dosage is defined with a certain width. For instance, products whose dosage are described as “X mg or Y mg depending on the condition” or “X mg in the usual case and Y mg in case of inadequate effect” were classified as “dose in range.” With respect to “upward/downward dose adjustment”, products with descriptions such as “dose may be adjusted” or “dose may be reduced” according to the patient’s condition were classified as “upward/downward dose adjustment.”
3. Clinical data package. Five factors, “orphan drugs,” “bridging strategy or multi-regional clinical trial,” “dose finding study,” “lower dose in pivotal study,” and “safety concern” were investigated. “Dose finding study” was defined as a study to examine efficacy and safety comparing two or more fixed dosages. For instance, products for which phase II clinical trials using only one dose of MTD or flexible dose were conducted were not classified as “dose finding study.” In “lower dose in pivotal study”, a pivotal study basically means phase III clinical trial, and exceptionally concerning the products such as antineoplastic agents for which phase III clinical trials are not conducted before approval; it means the latest phase clinical trial. When more than one phase III clinical trials were conducted, the products for which doses lower than the approved dose was examined in any of the studies were classified as “lower dose in pivotal study.” “Safety concern” was defined as the case for which adverse effects were taken into account in the recommended dose selection of a product, and the products with descriptions concerning adverse effects in the dose selection in the review reports or CTDs were classified as “safety concern.”
4. Post-marketing requirement. Products with the requirement for conducting post-marketing clinical studies or all case surveys were defined as “approval conditions.”

## Analyses

We conducted univariate and multivariate logistic regression analyses using “lower-dose prescription drugs” as a response variable and the 10 factors mentioned above as exploratory variables. A significant association was defined at p value < 0.1 in the univariate analysis, and all the associated variables were incorporated into the multivariate model; when a strong association (Cramér's V > 0.5) was identified between the selected explanatory variables in the univariate analysis, only one of the factors was selected to be included in the multivariate analysis. In the multivariate analysis, a statistically significant association was defined at p value < 0.05. The analyses were performed using StatsDirect (StatsDirect LTD., Cheshire, UK).

## Results

### Drugs examined

**Fig 1:**
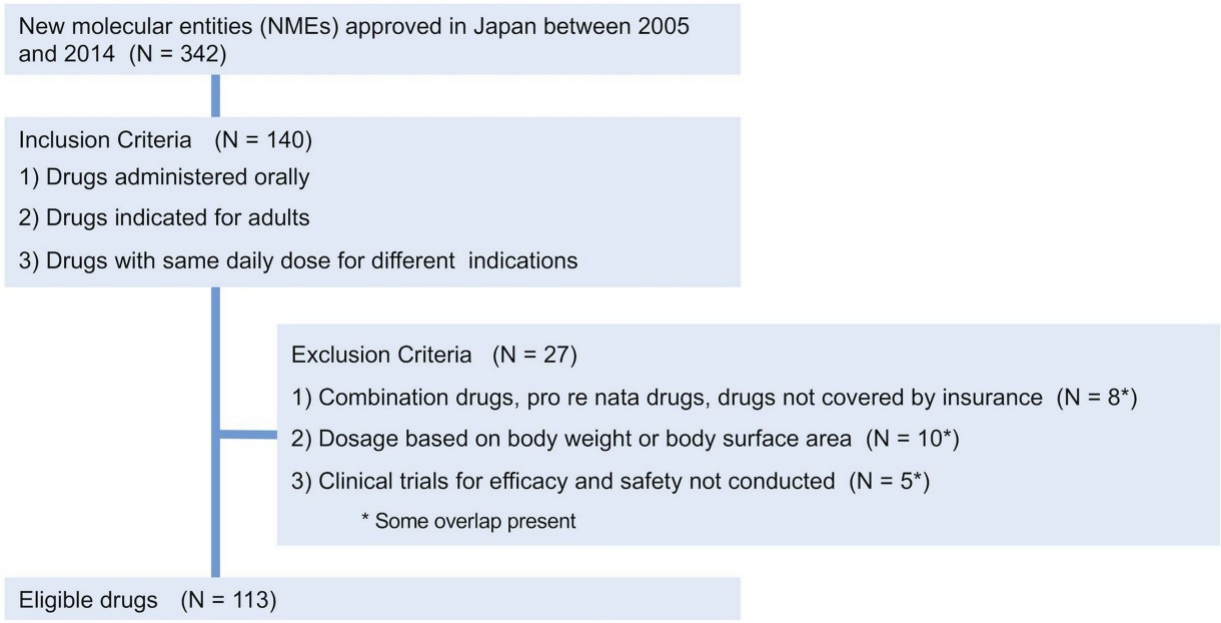
Flowchart representing the selection of drugs to be investigated.

From a total of 342 pharmaceutical products approved as NMEs in Japan between 2005 and 2014, we selected 140 products that fulfilled all the inclusion criteria. After excluding 27 products, a dataset of 113 products was created (Fig 1 and S1 Table).

### Lower-dose prescription drugs

Twenty-seven of the 113 (23.9%) investigated products were identified as “lower-dose prescription drugs.” The number and percentage of prescriptions by daily dose category in each database for the 27 products are shown in Table 1. Although there were a few differences, prescription trends were roughly similar between the two databases.

**Table 1:** List of the lower-dose prescription drugs.

Drugs	Categories of daily dose (mg)		Prescriptions in the database				Lower-dose prescriptions	
			MDV		JammNet		MDV	JammNet
			Number	Percent	Number	Percent	Percent	Percent
Afatinib	DSP	≥ 20, < 40	6,572	52.5%	171	61.1%	52.5%	61.1%
	AD	≥ 40, ≤ 50	5,716	45.6%	109	38.9%		
		> 50	241	1.9%	0	0.0%		
Atovaquone		< 1500	1,127	13.4%	68	34.3%	13.4%	34.3%
	AD	1500	7,148	85.0%	129	65.2%		
		> 1500	133	1.6%	1	0.5%		
Axitinib		< 4	252	3.9%	0	0.0%	44.7%	23.4%
	DSP	≥ 4, < 10	2,664	40.8%	32	23.4%		
	AD	≥ 10, ≤ 20	3,575	54.8%	105	76.6%		
		> 20	33	0.5%	0	0.0%		
Bosentan		< 125	1,086	13.4%	28	13.4%	56.7%	52.6%
	AID	≥ 125, < 250	3,497	43.3%	82	39.2%		
	AMD	250	3,424	42.4%	99	47.4%		
		> 250	72	0.9%	0	0.0%		
Bosutinib		< 400	356	35.5%	63	77.8%	55.7%	100.0%
	DSP	≥ 400, < 500	203	20.2%	18	22.2%		
	AD	≥ 500, ≤ 600	419	41.8%	0	0.0%		
		> 600	25	2.5%	0	0.0%		
Clozapine	AID	≥ 12.5, < 200	981	20.6%	115	36.9%	20.6%	36.9%
	AMD	≥ 200, ≤ 600	3,768	79.1%	197	63.1%		
		> 600	14	0.3%	0	0.0%		
Crizotinib		< 250mg	349	18.7%	0	0.0%	31.0%	10.7%
	DSP	≥ 250, < 500	228	12.2%	3	10.7%		
	AD	500	1,257	67.5%	25	89.3%		
		> 500	29	1.6%	0	0.0%		
Eplerenone		< 50	45,147	38.2%	1,658	33.4%	38.2%	33.4%
	AD	≥ 50, ≤ 100	72,712	61.6%	3,301	66.5%		
		> 100	204	0.2%	4	0.1%		
Ferric citrate		< 1500	17,078	52.4%	746	40.3%	52.4%	40.3%
	AD	≥ 1500, ≤ 6000	15,482	47.5%	1,104	59.7%		
		> 6000	2	0.0%	0	0.0%		
Gabapentin		< 200	148	0.7%	7	0.8%	68.3%	67.8%
	IDSP	≥ 200, < 300	2,973	13.7%	130	15.1%		
	MDSP	≥ 300, < 600	4,940	22.8%	195	22.7%		
	AID	≥ 600, < 1200	6,733	31.1%	250	29.1%		
	AMD	≥ 1200, 2400	6,782	31.3%	277	32.2%		
		> 2400	74	0.3%	0	0.0%		
Gabapentin Enacarbil		< 300	1	0.0%	0	0.0%	48.5%	46.4%
	DSP	≥ 300, < 600	1,051	48.5%	173	46.4%		
	AD	600	1,095	50.5%	200	53.6%		
		> 600	22	1.0%	0	0.0%		
Imidafenacin		< 0.2	22,924	34.7%	888	31.0%	34.7%	31.0%
	AD	≥ 0.2, ≤ 0.4	43,043	65.2%	1,973	69.0%		
		< 0.4	45	0.1%	0	0.0%		
Maraviroc	DSP	≥ 150, < 600	28	62.2%	0	0.0%	62.2%	0.0%
	AD	600	17	37.8%	5	100.0%		
Memantine		< 5	217	0.2%	0	0.0%	50.3%	40.0%
	AID	≥ 5, <10	25,954	18.4%	187	12.4%		
	DSP	≥ 10, < 20	44,974	31.8%	414	27.5%		
	AMD	20	68,336	48.3%	887	59.0%		
		> 20	1,951	1.4%	15	1.0%		
Miglustat	DSP	≥ 200, < 600	3	37.5%	0	NA	37.5%	NA
	AD	600	1	12.5%	0	NA		
		> 600	4	50.0%	0	NA		
Nilotinib		< 400mg	1,375	24.0%	2	1.5%	38.8%	18.2%
	DSP	≥ 400, < 600	849	14.8%	22	16.7%		
	AD	≥ 600, ≤ 800	3,473	60.5%	108	81.8%		
		> 800mg	42	0.7%	0	0.0%		
Pancrelipase		< 1800	29,220	45.3%	636	42.0%	45.3%	42.0%
	AD	1800	33,802	52.4%	854	56.4%		
		> 1800	1,518	2.4%	24	1.6%		
Pazopanib	DSP	> 200, < 800	2,521	57.5%	48	64.0%	57.5%	64.0%
	AD	800	1,795	41.0%	27	36.0%		
		> 800	66	1.5%	0	0.0%		
Pirfenidone		< 600	322	3.4%	1	1.1%	82.8%	72.6%
	AID	≥ 600, < 1200	3,420	36.6%	32	33.7%		
	DSP	≥ 1200, < 1800	3,991	42.7%	36	37.9%		
	AMD	1800	1,545	16.5%	26	27.4%		
		> 1800	58	0.6%	0	0.0%		
Regorafenib		< 80	142	3.6%	6	7.1%	68.7%	56.5%
	DSP	≥ 80, < 160	2,577	65.1%	42	49.4%		
	AD	160	1,210	30.6%	37	43.5%		
		> 160	28	0.7%	0	0.0%		
Ropinirole		< 0.75	386	6.2%	4	4.5%	32.9%	21.6%
	AID	≥ 0.75, < 3	1,676	26.7%	15	17.0%		
	AMD	≥ 3, ≤ 15	4,203	67.0%	69	78.4%		
		> 15	8	0.1%	0	0.0%		
Rufinamide		< 400	22	4.9%	0	0.0%	71.2%	31.0%
	AID	≥ 400, < 1800	300	66.4%	9	31.0%		
	AMD	≥ 1800, ≤ 3200	127	28.1%	20	69.0%		
		> 3200	3	0.7%	0	0.0%		
Sorafenib	DSP	≥ 200, < 800	10,170	77.2%	54	72.0%	77.2%	72.0%
	AD	800	2,926	22.2%	21	28.0%		
		> 800	81	0.6%	0	0.0%		
Telaprevir		< 2250	207	98.1%	0	NA	98.1%	NA
	AD	2250	3	1.4%	0	NA		
		> 2250	2	0.9%	0	NA		
Topiroxostat		< 40	1,542	14.6%	285	16.9%	92.3%	95.4%
	AID	≥ 40, < 120	8,204	77.7%	1,322	78.5%		
	AMD	≥ 120, ≤ 160	799	7.6%	77	4.6%		
		> 160mg	18	0.2%	0	0.0%		
Varenicline		< 0.5	1	0.0%	0	0.0%	7.8%	40.4%
	AID	≥ 0.5, < 1	78	0.9%	815	19.4%		
	DSP	≥ 1, < 2	614	7.0%	883	21.0%		
	AMD	2	5,436	61.5%	2,506	59.6%		
		> 2	2,705	30.6%	2	0.0%		
Vorinostat		< 300	23	29.5%	0	NA	92.3%	NA
	DSP	≥ 300, < 400	49	62.8%	0	NA		
	AD	400mg	6	7.7%	0	NA		

AD: approved dose, AID: approved initial dose, AMD: approved maintenance dose, DSP: dose for special population, IDSP: initial dose for special population, MDSP: maintenance dose for special population.

**Fig 2:**
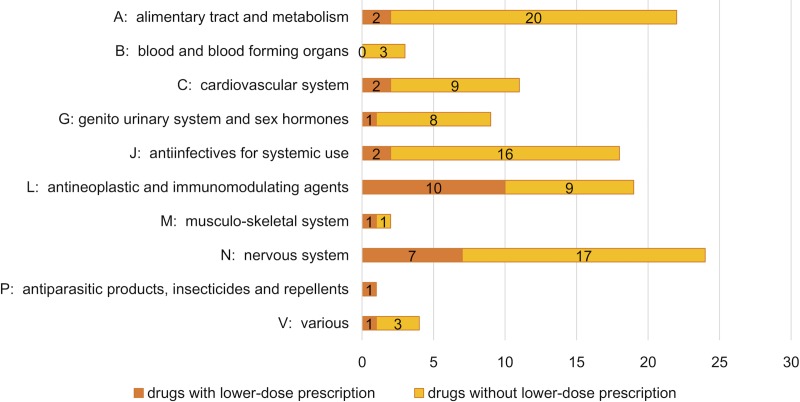
Number of drugs with or without lower-dose prescriptions according to the ATC classification. ATC: Anatomical Therapeutic Chemical.

The proportions of lower-dose prescription products classified based on the ATC code first level, anatomical main group, are shown in Fig 2. A relatively large number of products classified as “L; antineoplastic and immunomodulating agents” and “N; nervous system” was identified as “lower-dose prescription drugs,” 10 and 7 products, respectively.

**Fig 3:**
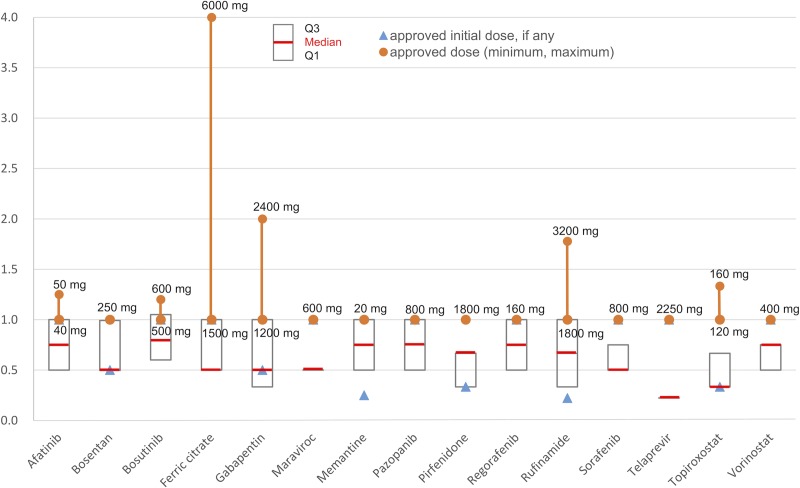
Median and quartile points of frequency distribution of prescribed dosage standardized by the minimum approved dose. The box plots show the median and quartile points of the prescribed daily dose. The red lines indicate the median. The blue triangles indicate the approved initial dose (different from the maintenance dose), the orange circles indicate the approved maintenance dose and the orange full lines indicate the range (if the initial dose is not set, the approved dose is the same as the approved maintenance dose), and the numbers in black indicate the real minimum and maximum of the approved maintenance doses.

Regarding the distribution of daily dose in the database of MDV, the median daily dose was less than the approved dose (or the approved maintenance dose if the initial dose is set) for 15 products. For these 15 products, the median and quartile points of the prescribed daily dose standardized by the minimum approved dose (the minimum approved dose equal to 1) are shown in Fig 3 and S2 Table. The data clearly indicated that the prescription doses in the actual situation were considerably lower than the approved dose.

### Univariate and multivariate analyses

The results of the univariate analysis are shown in Table 2.

**Table 2:** Results of the univariate analysis.

Parameter		Rate of lower-dose prescription drugs		Odds ratio	(95% CI)	p value
**ATC classification**						
Antineoplastic agents	Yes	9/12	75.0%	13.83	(3.40–56.24)	< 0.001
	No	18/101	17.8%			
**Detailed statement of the approved dosage**						
Dose range^a^	Yes	19/67	28.4%	1.88	(0.74–4.76)	0.183
	No	8/46	17.4%			
Maintenance dose different from the initial dose	Yes	9/10	90.0%	42.50	(5.06–356.79)	< 0.001
	No	18/103	17.5%			
Upward/downward dose adjustment^b^	Yes	9/15	60.0%	6.67	(2.10–21.11)	0.001
	No	18/98	18.4%			
**Clinical data package**						
Orphan drugs	Yes	9/28	32.1%	1.76	(0.68–4.55)	0.241
	No	18/85	21.2%			
Bridging strategy or multi- regional clinical trial	Yes	8/23	34.8%	1.99	(0.74–5.40)	0.175
	No	19/90	21.1%			
Dose finding study^c^	Yes	14/85	16.5%	0.23	(0.09–0.58)	0.002
	No	13/28	46.4%			
Lower dose in pivotal study^d^	Yes	8/16	50.0%	4.11	(1.37–12.34)	0.012
	No	19/97	19.6%			
Safety concern^e^	Yes	18/53	34.0%	2.91	(1.17–7.23)	0.021
	No	9/60	15.0%			
**Post-marketing requirement**						
Approval conditions^f^	Yes	11/34	32.4%	1.88	(0.76–4.65)	0.170
	No	16/79	20.3%			

CI: confidence interval.

^a^The approved dosage is within a certain width.

^b^Products with description such as “dose may be adjusted” or “dose may be reduced” according to the patient’s condition in the approved dosage

^c^A study to examine the efficacy and safety comparing two or more fixed dose was conducted for the product.

^d^Lower dose was examined in phase III study or latest phase study before approval.

^e^Products for which adverse effects were considered in dose selection.

^f^Requirement for conducting post-marketing clinical study or all-case survey.

Six factors, namely, “antineoplastic agents,” “maintenance dose different from initial dose,” “upward/downward dose adjustment,” “dose finding study,” “lower dose in pivotal study,” and “safety concern,” were selected as candidates for the multivariate analysis. A strong association between “antineoplastic agents” and “dose finding study” (Cramér's V = 0.534) was identified, and we selected five factors excluding “dose finding study” as exploratory variables for the multivariate analysis. The results of the multivariate analysis revealed that “antineoplastic agents,” “maintenance dose different from the initial dose,” and “upward/downward dose adjustment” significantly associated with “lower-dose prescription drugs” (p < 0.05) (Table 3).

**Table 3:** Results of the multivariate analysis.

Parameter	Odds ratio	(95% CI)	p value
Antineoplastic agents	14.44	(2.73–76.51)	0.002
Maintenance dose different from the initial dose	79.82	(8.49–750.26)	< 0.001
Upward/downward dose adjustment^a^	6.05	(1.33–27.59)	0.020
Lower dose in pivotal study^b^	2.20	(0.42–11.39)	0.349
Safety concern^c^	1.54	(0.42–5.61)	0.514

^a^Products with description such as “dose may be adjusted” or “dose may be reduced” according to a patient’s condition in the approved dosage.

^b^Lower dose was examined in phase III study or latest phase study before approval.

^c^Products for which adverse effects were considered in the dose selection.

## Discussion

We have clarified two aspects in the present study. First, with respect to the actual situation of lower-dose prescriptions in clinical use, we identified 27 products (23.9%) as “lower-dose prescription drugs.” This indicates that the dose of approximately one-third or more prescriptions was lower than the approved dose among the 113 products approved in Japan between 2005 and 2014. We believe this borderline can be one of the criteria for reconsidering the approved dosage to be applicable to a greater number of patients. This finding is consistent with the results reported previously, that is, approximately 20% of 449 NMEs approved between 1980 and 1999 in the United States were subjected to dose change after approval, with approximately 80% of the changes involving switch to a lower dose [1], and approximately 60% of the products whose WHO DDD was changed between 1982 and 2000 was changed to a lower dose [2]. The present study highlighted prescriptions at doses lower than the approved dose in the actual post-marketing scenario.

Second, the results showed that the factors related to ATC classification and the detailed statement of the approved dosage significantly influenced lower-dose prescriptions, but those related to clinical data package and post-marketing requirement did not. As the results indicated that “antineoplastic agents” a factor of ATC classification was strongly related to “dose finding study” a factor of clinical data package, we thought that priority was given to satisfying the clinical needs in the development process and that dose selection was not made based on sufficient study results. This is in accord with the fact that the U.S. Food and Drug Administration and some relevant scientific societies have been discussing the method of dose selection in the clinical development and dose optimization post-marketing for oncology drugs [9, 13].

As for the detailed statement of the approved dosage, drugs with “maintenance dose different from the initial dose” and “upward/downward dose adjustment” were found to be prescribed at a lower dose. For several products with the initial dose or titration dose, gradual dose titration from the initial low dose is recommended in the product label to ensure initial tolerability. It is reasonable to assume that the dose of such products cannot be increased to an effective dose due to adverse effects or may not be increased further due to the clinical judgment of sufficient efficacy. That is, products with “maintenance dose different from the initial dose” and “upward/downward dose adjustment” might have a large variation in the responsiveness among patients, which indicates that dose adjustment for individual patients is difficult.

On the contrary, previous reports have suggested that the efficacy at low doses is not sufficiently studied during the development period because phase III trials mainly focus on the confirmation of efficacy; this is one of the reasons for the use of a lower dose of a drug post-marketing [1, 6]. In the present study, although there was no statistically significant difference, several products of “lower dose in pivotal study” corresponded to “lower-dose prescription drugs.” This tendency suggests that when a dose lower than the approved dose is used in phase III trials, even if the dose was not ultimately selected as the approved dose, the lower dose might be used as an effective dose in dose titration for individual patients because of its clinical efficacy to a certain degree.

Recently, in order to respect the voice of individual patients, Patient-Report Outcome (PRO) has been utilized in clinical trials, and the results are described in the product label for some medicines [14–15]. Although we did not collect the data regarding the use of PRO in the clinical trials in the present study, the movement to utilize PRO including Patient-Report Outcomes version of Common Terminology Criteria for Adverse Event (PRO-CTCAE) for the evaluation of adverse events in antineoplastic agents [16] may contribute to determining the optimal dose in the clinical development process [17].

Prescription of drugs at a lower dose, unlike dose prescriptions higher than the approved dose, is likely to be accepted as an optimization strategy for individual patients under a physician’s discretion. Although there are reports that recommend the use of doses lower than the approved dose for the elderly population or for reducing adverse effects [3–6], the optimized dose should be indicated in the product label and relevant information should be provided to ensure that anyone can use the drug at the optimal dose.

In the present study, we also investigated relevant factors from the perspective of clinical data package and post-marketing requirement. Although the proportion of drugs corresponding to “lower-dose prescription drugs” was marginally high with respect to “safety concern” and “approval conditions,” these drugs were not identified to be related to “lower-dose prescription drugs.” This shows the limitation in predicting lower-dose prescriptions for use in various actual post-marketing situations from the results of clinical trials before approval, and also the possibility that the approved dose may not be identical to the optimal dose after marketing. Furthermore, it emphasizes the importance of reexamining the approved dosage that is applicable to a greater number of patients after marketing, if necessary.

In particular, we observed some products for which the percent of deviated prescriptions exceeded 90%. Thus, the clinically used dosage in the post-marketing phase of a drug should be monitored, and it is important to recognize the recommended dosage, which is applicable to a greater number of patients without showing lack of efficacy. We believe that the Real-World Data (RWD) utilized more in recent years would help resolve the deviation from the approved dose and lead to a prompt delivery of information on the optimized dose for each pharmaceutical product. Real-world evidence accumulates clinical evidence based on the actual use of drugs after marketing and will therefore, compensate for the limited information obtained before approval [18, 19]. We can utilize RWD to identify patient backgrounds with lower-dose prescriptions and plan post-marketing clinical trials to clarify benefit/risk balance using lower dose for those specific populations, if necessary.

Limitations to our study were that the information of individual patients’ background to whom lower doses of drugs were prescribed was not collected and that the prescribed daily doses were not related to individual patients. The databases included prescription data of both elderly and non-elderly populations, and it was not clear whether the lower-dose prescriptions were mainly for the elderly population. However, we understand that the data show a general picture of prescriptions for all adult populations. In addition, the fact that parenteral preparations (e.g., injections and inhalations) were not included in the drugs examined is another limitation. We need to interpret the results with caution considering these limitations. Further studies are needed to clarify situations and backgrounds related to lower-dose prescriptions.

## Conclusions

To the best of our knowledge, the present study is the first to elucidate the actual situation of lower-dose prescriptions and the influencing factors. The results suggest the limitations in determining a dose applicable to the majority of patients for various post-marketing usages, emphasizing the importance of reexamining the optimal dose, if necessary. We believe that the utilization of RWD could be of help in this regard.

## Supporting information

S1 Table Dataset of factors related to the therapeutic area, detailed statement of the approved dosage, clinical data package, and post-marketing requirement for each eligible drug. For each factor, “1” indicates yes, “0” indicates no. “Lower-dose prescription” drugs were products whose percentage of prescriptions corresponding to the category of doses lower than the approved dose, or the approved maintenance dose if the initial dose is set, was ≥ 30% in the database of either MDV or JammNet. The definition of factors related to the therapeutic area, detailed statement of the approved dosage, clinical data package, and post-marketing requirement was the same as those in Table 2.
(XLSX)

S2 Table Median and quartile points of the prescribed daily dose. (XLSX)
